# Estimation of genetic diversity in viral populations from next generation sequencing data with extremely deep coverage

**DOI:** 10.1186/s13015-016-0064-x

**Published:** 2016-03-11

**Authors:** Jean P. Zukurov, Sieberth do Nascimento-Brito, Angela C. Volpini, Guilherme C. Oliveira, Luiz Mario R. Janini, Fernando Antoneli

**Affiliations:** Departmento de Medicina, Escola Paulista de Medicina (EPM), Universidade Federal de São Paulo (UNIFESP), São Paulo, Brazil; Departamento de Microbiologia, Imunologia e Parasitologia, Escola Paulista de Medicina (EPM), Universidade Federal de São Paulo (UNIFESP), São Paulo, Brazil; Departmento de Informática em Saúde, Escola Paulista de Medicina (EPM), Universidade Federal de São Paulo (UNIFESP), São Paulo, Brazil; Laboratório de de Biocomplexidade e Genômica Evolutiva, Escola Paulista de Medicina (EPM), Universidade Federal de São Paulo (UNIFESP), São Paulo, Brazil; Departamento de Microbiologia e Imunologia Veterinária, Universidade Federal Rural do Rio de Janeiro (UFRRJ), Rio de Janeiro, Brazil; Genomics and Computational Biology Group, Centro de Pesquisas René Rachou (CPqRR), Fundação Oswaldo Cruz (FIOCRUZ), Belo Horizonte, Brazil

**Keywords:** Viral diversity, Bayesian inference, Dirichlet distribution

## Abstract

**Background:**

In this paper we propose a method and discuss its computational implementation as an integrated tool for the analysis of viral genetic diversity on data generated by high-throughput sequencing. The main motivation for this work is to better understand the genetic diversity of viruses with high rates of nucleotide substitution, as HIV-1 and Influenza. Most methods for viral diversity estimation proposed so far are intended to take benefit of the longer reads produced by some next-generation sequencing platforms in order to estimate a population of haplotypes which represent the diversity of the original population. The method proposed here is custom-made to take advantage of the very low error rate and extremely deep coverage per site, which are the main features of some neglected technologies that have not received much attention due to the short length of its reads, which precludes haplotype estimation. This approach allowed us to avoid some hard problems related to haplotype reconstruction (need of long reads, preliminary error filtering and assembly).

**Results:**

We propose to measure genetic diversity of a viral population through a family of multinomial probability distributions indexed by the sites of the virus genome, each one representing the distribution of nucleic bases per site. Moreover, the implementation of the method focuses on two main optimization strategies: a read mapping/alignment procedure that aims at the recovery of the maximum possible number of short-reads; the inference of the multinomial parameters in a Bayesian framework with smoothed Dirichlet estimation. The Bayesian approach provides conditional probability distributions for the multinomial parameters allowing one to take into account the prior information of the control experiment and providing a natural way to separate signal from noise, since it automatically furnishes Bayesian confidence intervals and thus avoids the drawbacks of preliminary error filtering.

**Conclusions:**

The methods described in this paper have been implemented as an integrated tool called *Tanden* (Tool for Analysis of Diversity in Viral Populations) and successfully tested on samples obtained from HIV-1 strain NL4-3 (group M, subtype B) cultivations on primary human cell cultures in many distinct viral propagation conditions. *Tanden* is written in C# (Microsoft), runs on the Windows operating system, and can be downloaded from: http://tanden.url.ph/.

## Background

Viruses with RNA genomes are recognized to generate particularly mutant-rich populations called *quasispecies*. The genetic heterogeneity characteristic of viral quasispecies is largely due to high mutational rates combined with an elevated population size [1]. Human immunodeficiency virus 1 (HIV-1), as an example, has a mean substitution rate of order 10^−5^ per nucleotide position [2]; that is by far higher than those of cellular organisms [3, 4] and assures a constant viral mutant production.

Next-generation sequencing (NGS) platforms have been used mainly for the de novo sequence assembly of viruses. However, more recently, new interest arose in re-sequencing known virus genomes using NGS to study the diversity of viral populations. All NGS platforms produce short segments of DNA, called *reads*, which provide only imperfect and incomplete information about the structure of the viral population. Sequencing errors and length of reads are factors that must be taken into account in the analysis of data obtained from NGS viral quasi-species. In addition, reverse transcription and PCR amplification are procedures prone to errors. The impact of these errors on studies of viral diversity could be huge (see below), therefore one wants to separate true genetic variation from methodological noise and if both are of the same order of magnitude the task becomes virtually impossible.

Regarding the development of tools to estimate genetic diversity of viral populations (total number of genetic characteristics in a viral ensemble), the most commonly used NGS platforms are the 454™ (Life Sciences/Roche)—since Roche’s announced in October 2013 it will shutdown the 454™ platform [5] its use has been fading—and the Illumina™ (Solexa), mainly due to their capacity to produce longer reads. The ability to produce relatively long sequences favors the development of methods aiming at the haplotype (the genetic variants in a viral population) reconstruction of the representative viral particles in population [6, 7]. However, the propagation of sequencing errors is a serious problem in these methods, requiring the development of procedures for error correction, which my introduce unwanted biases. In general, the fraction of wrong reads increases with the error rate per base and the average length. The expected proportion of reads with at least one sequencing error as a function of the error rate per base *ε* and the average length *L* of reads is given by the relation 1 − (1 − *ε*)^*L*^ [8]. As the estimated error rate of 454™ is about 0.1–0.5 % and Illumina™ error rates are in the range of 0.1–1 % [9], with an average length of reads from 400 bp up to 1000 bp, the proportion of reads with at least one error is in the range 35–90 %. The platform SOLiD™ (Life Technologies), for instance, is at the other end of the spectrum. With reads of short length, of at most 50 bases (the main limitation for the construction of haplotypes) and estimated error rate of 0.06 % [9], the proportion of reads with at least one error is around 2 %. Recently, a different solution to the problem of sequencing errors has been proposed [10], based on the development of high-fidelity sequencing protocols [11].

A more serious challenge associated with the assembly of all possible haplotypes is the *NP*-*hardness* of the corresponding combinatorial optimization problems [12]. In fact, some approximate solution must be employed and a crucial hindering factor is the ratio between the size of the reads and the size of the genomic region being reconstructed. For instance, it has been reported [10] that short read lengths (less than 100 base pairs) dramatically inhibit reconstruction of genomes with more than 3400 bp, evidenced by the failing to produce any complete genome. Another major shortcoming of all existing methods for haplotype reconstruction is that they are unable to handle large insertions or deletions (indels), only very recently this problem seems to have been overcome [13].

As mentioned before, the ability of the other NGS platforms to produce relatively long sequences have been a great stimulus to the development of methods for building *haplotype representatives* of viral particles in the population and the vast majority of softwares for viral diversity estimation that have been proposed until very recently adopt this perspective [6]. The aim of this work is to propose a different approach to measure genetic diversity that does not demand any kind of length assumption on the short reads, but takes advantage of the low error rate and the high depth of coverage per site inherent to some NGS platforms. Therefore, we shall considerably depart from the most traditional developments aiming at haplotype reconstruction, since not every one has access to the NGS platforms appropriate for that purpose. Indeed, although the short length of the reads produced by these platforms essentially hinders haplotype reconstruction, it is possible to measure genetic diversity through probability distributions along the genome (one per site) and this approach is enhanced by the highly deep coverage provided by these NGS platforms.

A recent study [14] comparatively assessed the performance of some NGS platforms (including 454™ and Illumina™) and reported an average (range) coverage of ~23,000 reads (5000–47,000) for the Illumina™ and ~7000 reads (2000–22,000) for the 454™. We used the SOLiD™ platform and were able to achieve an average (range) coverage of ~50,000 reads (10,000–150,000), for instance (see Fig. 2). In addition, the low error rate of 0.06 % provided by the SOLiD™ platform virtually eliminates the necessity of any error correction procedure. Instead, we use the estimated probability distributions to separate signal from noise.

The first step in nucleotide sequence analysis is read mapping/alignment. This is important for many bioinformatics applications, as exemplified by nucleic acid conformational structure prediction and phylogeny studies [15, 16]. As expected, this is also an important aspect for NGS data analysis involving all the different platforms as Ion Torrent™ (Life Technologies), SOLiD™, 454™ and Illumina™ [17, 18] and others. Nowadays, users can choose from a panoply of tools for mapping and indexing NGS reads, available on-line and for download. MAQ (Mapping and Assembly with Qualities) [19], BWA (Burrows–Wheeler alignment tool) [20], BFAST (Blat-like fast accurate search tool) [21], Bowtie [22] and MOSAIK [23] are examples of such alternatives. Those tools allow the fast mapping and alignment of reads belonging to genomes up to 10^9^ bp in length [20, 24, 25].

After the read mapping is finished, the following step consists in the choice of a strategy for statistical inference. There is a wide variety of methods depending on the scope and the goals of the analysis: (1) consensus generation, (2) *single nucleotide variant* (SNV), also called *single position diversity estimation*, (3) *local diversity estimation* and (4) read graph-based haplotype reconstruction, also known as *global diversity estimation*, see [8, 26] for a thorough explanation of these concepts. Existing tools for genetic diversity evaluation of viral NGS sequences, intended for 454™ and Illumina™ platforms [8, 26–33], are based on several techniques aiming at haplotype reconstruction [30, 34–39].

In order to estimate the genetic diversity without resorting to haplotype reconstruction, we propose to represent the genetic diversity of a sample population through a family of multinomial probability distributions indexed by the sites of the virus genome, each one representing the distribution of nucleic bases per site. Moreover, the inference of the multinomial parameters is done in a Bayesian framework using smoothed Dirichlet estimation inspired by a method for modeling text data [40].

Inference of multinomial parameters is a challenging problem in statistics. For the simplest case, i.e., the Bernoulli model or binomial estimation, the history traces back to Thomas Bayes [41]. Karl Pearson [42] called this seemingly simple problem the “fundamental problem of practical statistics”. In the frequentist context the problem is called “interval estimation of a binomial proportion” and there is a textbook solution based on a confidence interval for this problem, which however has several drawbacks [43]. In both frameworks the frequency of occurrence of a category plays a crucial role, leading to the “sufficient statistics” in the Bayesian context and the “estimator of proportion in a sample” in the frequentist context.

The choice of a Bayesian framework is motivated by two features that are not present in the frequentist framework: (1) it allows one to obtain conditional (posterior) probability distributions for the multinomial parameters and thus interpret the point estimates as probabilities—this interpretation conceptually incorrect when applied to pure relative frequencies (which is not the same thing as adopting frequentist framework)—even though the law of large numbers implies that they converge to the point estimates obtained from the Dirichlet distributions when the number of observations goes to infinity; (2) on may take into account the prior information of the control experiment (whose genome sequence is known) within the inference of a posterior experimental condition by means of *Bayes’ formula* and thus relate two temporally connected events. Finally, it provides a natural way to separate signal from noise through credible (or Bayesian confidence) intervals—another problem with the use of pure relative frequencies is that it is not possible to associate “error bars” to them. Therefore, in our aproach, the errors introduced during the sequencing process are not filtered before the inference, but after it, when we identify the relevant signal—this allows us to avoid the drawbacks of preliminary error filtering [10, 44].

In summary, we sought to build an analysis platform suitable to address the problem of estimation of the populational genetic diversity of RNA viruses. Due to high mutational rates and accelerated replicative kinetics, RNA viruses constitute ensembles of variants, known as *quasispecies*, which, instead of a collection of viral particles, behave as a single and coherent organism which is act on by the host’s pressures [45]. Furthermore, our mathematical and computational approach allows for a better understanding of virtually all the viral diversity present in a clinical sample. By saying this, we mean that our method gives the user an idea about the real structure of a viral population contained in a clinical sample at a given time. When a quasispecies population is challenged by selective pressures, it responds as a sole organism due to the mutational link between the genetic variants it contains. Following this train of thoughts, at any given moment this distribution of mutants may bear variants with resistance mutations (which could minimize therapeutic success) or virus with genomic compositions that were sufficiently close to therapeutical resistance. In this manner, as far as clinical applications are concerned, our method offers an opportunity to the clinician to observe the entire viral mutational landscape in a clinical sample. This comprehensive view could help the clinician when deciding the best therapeutic approach.

Based on the aforementioned assumptions and that a NGS platform generates an extremely high number of reads of short length allowing for a deep and extensive coverage of the data, and with a very low error rate, we propose an approach to the estimation of genetic diversity of viral populations that does not make requirements on the form of the sequenced data (such as [10], which works only with Illumina™) and does not assume any statistical model for filtering errors [8]. Despite its apparent mathematical and computational involvement the approach proposed here is one of the simplest conceptually correct possible choices.

Unfortunately, we could not find any other method or software in the literature, which uses a similar form to represent the viral genetic diversity as a family of distributions indexed by the genome and does not need sufficiently long reads—all proposals in the literature that we were able to find are aimed at haplotype reconstruction and require longer reads (more than 100 bases). Any attempt to make comparison between such different approaches would be misleading, therefore it is not our aim here to make the point if the method presented here is an improvement over (non-)existing similar ones. In fact, we believe that the approach proposed here should not be considered alternative or rival, but complementary, to haplotype reconstruction.

## Implementation

**Fig. 1:**
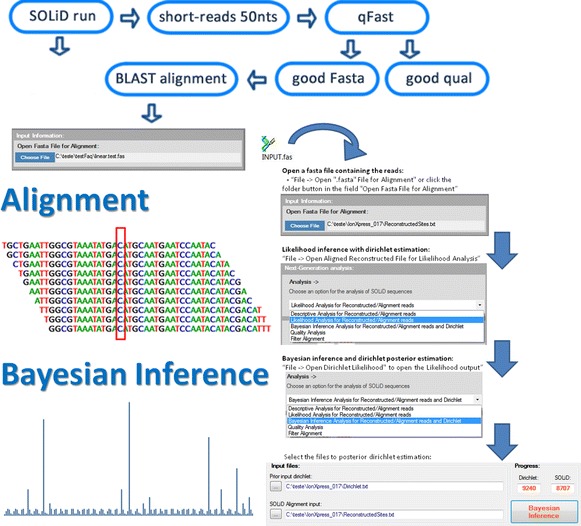
Flowchart containing the main steps of our method

Here we describe the main steps of our method. There are two stages, the first is the read mapping/alignment and the second is the nucleic bases inference (see flow-chart in Fig. 1). The method presented here works, in principle, with data generated in any NGS machine, as long it is (converted to and) stored in the *FASTA file format*. Even several distinct outputs from different platforms (with distinct read lengths) may be combined into one file and used as input.

## Experimental procedure and preparation

The computational tool developed here assumes that there is one, or more, different viral propagation situations (such as, varying the cellular activation status, the co-receptor usage and the target cells, etc.), all of which have the same viral population as infecting source (see Table 1). A sample from the viral population prior to any infection experiment must be sequenced and the raw sequence data obtained will be referred to as the *control experiment*. The importance of the control experiment is that it will be used to infer the noise of the system, since it is essentially a clonal population. The raw sequence data obtained from samples extracted from infected cells, after a fixed number of replication cycles, will be collectively called e*xperimental conditions*.

**Table 1:** Summary of sequence data analyzed

Sequenced data	Reads	Nucl.	Mapped (%)	Time (hour)
HIV1—non-stimulated CD4/R5	1.11 × 10^7^	5.53 × 10^8^	91.81	34.88
HIV2—stimulated CD4/R5	1.04 × 10^7^	5.19 × 10^8^	90.84	34.18
HIV3—non-stimulated CD4/X4	1.10 × 10^7^	5.50 × 10^8^	91.22	29.05
HIV4—stimulated PBMC/X4	9.41 × 10^6^	4.71 × 10^8^	91.14	13.63
HIV5—stimulated PBMC/R5	1.14 × 10^7^	5.73 × 10^8^	90.49	24.09
HIV6—non-stimulated PBMC/X4	1.12 × 10^7^	5.62 × 10^8^	91.42	27.54
HIV7—non-stimulated PBMC/R5	1.19 × 10^7^	5.94 × 10^8^	93.27	15.34
HIV8—control (pNL4-3kfs)	1.01 × 10^7^	5.05 × 10^8^	93.10	30.14
Total	8.65 × 10^7^	4.33 × 10^9^	91.67	208.85

The first column lists all the experimental conditions and the control experiment that where sequenced, the second column (Reads) displays the number of reads sequenced in each condition, the third column (Nucl.) displays the number of nucleotides in each condition, the fourth column (Mapped) displays the percentage of reads that have been mapped and the fifth column (Time) displays the time elapsed in each mapping procedure

Raw sequence data from the sequencing must be treated according to the standard procedures of the specific NGS platform [46] up to the generation of FASTA files, which are the standard type of input file adopted in our implementation.

The data used in this paper for testing the method is the subject of another publication [47], where the details of the experiments and the biological implications to the HIV replication are discussed. The method and the results described here do not depend on the experimental details and the results reported in [47].

## Read mapping/alignment

The main goal of this step is the mapping of reads with 50 nucleotides or more originating from the NGS platform to a database of reference sequences. The database may contain several sequences, which must be aligned amongst themselves. The read mapping is performed using a local executable of BLAST [48]. The criteria for retaining the reads are the following: (1) it must align at least 45 nucleotides and (2) have the lowest *e* value score. A first alignment attempt is made with sequences from reads in the forward sense; in case of no match, a second attempt with the reverse complementary sequence is performed. Moreover, since we are using several references, the output can, in principle, display the same number of matches as there are reference sequences. The criteria for the selection of the most suitable alignment option are the following (in this order): (1) the lowest *e* value score and (2) the lowest *Hamming distance* from the consensus sequence obtained from of the sequencing of the control experiment. The alignment strategy described above is set as default, but the user, according to some specific purposes or simply for increasing processing speed, can change some of its parameters. Finally, it is possible to create suitable reference databases for specific research purposes.

## Nucleic base estimation

The probability distributions of nucleic bases (A, T, C, G) at each position of the genome are estimated from the aligned data. In this respect, our approach may be classified as a *diversity estimation in single positions*. The idea is that at each position in the genome the probability distribution is given by a *multinomial distribution,* determined by four probabilities (*p*_A_, *p*_T_, *p*_C_, *p*_G_) satisfying *p*_A_ + *p*_T_ + *p*_C_ + *p*_G_ = 1. These conditional probabilities represent the fraction of the population that has each of the four associated nucleic bases at the corresponding site given the observed sequence data. Thus, one has a family of multinomial probability distributions indexed by the sites of the genome, where at each position the four probabilities (*p*_A_, *p*_T_, *p*_C_, *p*_G_) should be estimated from the data.

The Bayesian framework for the inference of categorical data is based on the notion of *conjugate prior,* which in the case of categorical data is given by the Dirichlet distributions [49, 50]. A *Dirichlet distribution* is characterized by a *n*-tuple of positive numbers ***α*** = (*α*_1_,…, *α*_*n*_) called *hyper*-*parameters*—however, unlike the multinomial parameters that must sum to one, the hyper-parameters are unconstrained. In our case, the Dirichlet distribution of each site is parametrized by the quadruple (*α*_A_, *α*_T_, *α*_C_, *α*_G_). The fact that the Dirichlet distribution is the conjugate prior of the multinomial distribution amounts to saying that the *Bayes’ formula* for the posterior distribution takes a very simple form in terms if the hyper-parameters: if (*α*_1_,…,*α*_*n*_) is a vector of hyper-parameters of a Dirichlet prior distribution and the counts of each of the *k* categories in an experiment are (*c*_1_,…, *c*_*n*_) then the posterior distribution is also a Dirichlet distribution with hyper-parameters (*α*_1_ + *c*_1_,…, *α*_*n*_ + *c*_*n*_). Within this context, the first step in the estimation of the probability distributions of nucleic bases consists in using the sequenced data from the *control experiment* as the input for the determination of prior hyper-parameters. Then, in the second step, one considers this distribution together with the sequenced data form the experimental conditions one uses *Bayes’ formula* to compute the *posterior hyper*-*parameters*.

A *n*-dimensional *Dirichlet distribution* is defined in by a smooth probability density function on the set Δ of *n*-dimensional multinomial distributions, which is parametrized as ${\rm\Delta_{n}} = \left\{ {\left( {p_{1} , \ldots ,p_{n - 1} } \right):p_{1} + \cdots + p_{n - 1} \le 1} \right\}$, here *n* is the number of distinct categories (states) that can observed and *p*_*k*_ is the probability of observing the *k*-th category, for *k* = 1,…, *n* with $p_{n} = 1 - p_{1} + \ldots + p_{{n - 1}}$. The *Dirichlet probability density* function is given by

$${\text{Dir}}\left( {\left. {p_{1} , \ldots ,p_{n - 1} } \right|\alpha_{1} , \ldots ,\alpha_{n} } \right) = \frac{1}{{\text{B}} (\varvec{\alpha})}\prod_{k} p_{k}^{{\alpha_{k} - 1}}$$

where B(***α***) is a normalizing factor defined in terms of the *gamma function* Γ as

$${\text{B}}(\mathbf{\alpha}) = \frac{\prod_{k} {\Upgamma (\alpha_{k} )}}{\Upgamma \bigg(\sum_{k} \alpha_{k} \bigg)}$$

for a vector ***α*** = (*α*_1_,…, *α*_*n*_). Note that the choice (*α*_1_,…, *α*_*n*_) = (1,…, 1) gives the uniform distribution (the *flat or uninformative prior*) on Δ_*n*_ with mass equal to the volume of $\Delta _{{\text{n}}} :{\text{B}}({{1}}, \ldots ,{\text{1}})\,{{ = 1/}}\Gamma ({\text{n}})\,{{ = 1/}}({\text{n}}{ - }{{1}})!$.

The Dirichlet hyper-parameters associated to the sequenced data from the control experiment represent the “noise” of the system and can be obtained by maximum likelihood estimation (MLE) through the Newton–Raphson method [51]. The *log*-*likelihood functiong* of the Dirichlet distribution is given by *g* = *N* log *L* and

$$\log \, L\left( {\alpha_{1} , \ldots ,\alpha_{n} \left| {p_{1} , \ldots ,p_{n} } \right.} \right) = \log \,\Upgamma \left( {\sum_{k} \alpha_{k} } \right) - \sum_{k} \log\, \Upgamma \left( {\alpha_{k} } \right) + \sum_{k} \left( {\alpha_{k} - 1} \right)\log \, p_{k} ,$$

where *N* is the *sample size* and log $p_{k} = 1/N \,{\sum_{j} \log \, p_{jk}}$ (*j* = 1,…, *N*, *k* = 1,…, *n*) is called the *sufficient statistics* associated to a sample of *n*-categorical vector observations {***p***_1_,…, ***p***_*N*_} of sample size *N*. Thus each vector ***p***_*j*_ = (*p*_*j*1_,…, *p*_*jn*_) has *n* components, each component *p*_*jk*_ is the frequency of the *k*th category at the *j*th sample.

The *Newton*–*Raphson method* for the maximum likelihood estimation of Dirichlet hyper-parameters amounts to the iteration of the following fixed-point scheme [49], which converges to the unique maximum value of *g*:

$$\varvec{\alpha}^{\text{new}} =\varvec{\alpha}^{\text{old}} + \left[ {H^{ - 1} \nabla g} \right]\left( {\varvec{\alpha}^{\text{old}} } \right),$$

where ***α***^new^ and ***α***^old^ are vectors of Dirichlet hyper-parameters. The function ∇*g*(***α***) is the *gradient vector* (derivative) of the log-likelihood function *g*, with components

$$\left[ {\nabla g\left(\varvec{\alpha}\right)} \right]_{k} = \Uppsi \left( {\sum_{k} \alpha_{k} } \right) - \Uppsi \left( {\alpha_{k} } \right) + \log p_{k} ,$$

where Ψ = (log Γ)′ is the *digamma function*. The function *H*^−1^(***α***) is the inverse of the *hessian matrix* of the log-likelihood function *g* and the product (*H*^−1^∇*g*)(***α***) = *H*^−1^(***α***)∇*g*(***α***) has components [(*H*^−1^∇*g*)(***α***)]_*k*_ given by

$$\begin{aligned} \left[ {\left( {H^{ - 1} \nabla g} \right)\left( {\boldsymbol\alpha} \right)} \right]_{k} & = \left( {\Uppsi^{\prime}( {\alpha_{k} })} \right)^{ - 1} \left( \left( {\nabla g} \right)_{k}\right. \\ &\quad + \left.{\left(\sum_{l} \frac{(\nabla g)_{l}}{\Uppsi^{\prime}(\alpha_{k})} \right)} \bigg/ \left(\frac{1}{\Uppsi^{\prime}\left(\sum_{l} \alpha_{l}\right)} - \sum_{l} \frac{1}{\Uppsi^{\prime}(\alpha_{l})}\right) \right),\end{aligned}$$

where Ψ′ is the *trigamma function* (*k*, *l* = 1,…, *n*). Several suggestions for the initialization step (that is, the initial value of ***α***^old^) of the iteration scheme described above have appeared in the literature [50, 52, 53]. The proposal of Ronning [53] is the most suitable for the modified iteration scheme adopted here.

Since we are dealing with a sparse estimation problem in the sense that one of the categories occur with much higher frequency that the other categories, we shall employ the *smoothed sufficient statistics* defined by introducing a small parameter *η* and setting *p*_*jk*_ = *M*_*jk*_*/M*, where *M*_*jk*_ is the number of occurrences of the *k*th category at the *j*th sample, *M* is total number of observations at the *j*th sample and *p*_*jk*_ = *η* if there is no occurrence of the *k*th category at the *j*th sample. The *smoothing parameterη* acts as “background noise” representing sequencing and PCR errors that can not be removed. However it can be suitably tuned in order to account for the true variability of the data. When this procedure is applied to the sequenced data from the control experiment (a “clonal” population) one would expect no diversity at all. However, that is not completely true and, in fact, even the sequenced data from the control experiment should display some variability (mainly due to sequencing errors). The smoothing parameter *η* should be at same (or smaller than) of the order of magnitude of expected error rate *ε*. In the smoothed version of the Newton–Raphson iteration scheme, Ronning’s initialization step is given by setting ***α***^old^ = (*η*,…, *η*).

The sufficient statistics is computed by a simple re-sampling procedure [54, 55] in order to generate sequences of categorical observations from the raw sequenced data, by randomly sampling nucleotides form each aligned position. Here, the imperfect clonality of the sequenced data from the control experiment is useful, since it ensure that the re-sampled ensemble has some variability, which is consistent with having a small non-zero smoothing parameter. The re-sampling procedure has one parameter that can be adjusted by the user: the relative size of observations given as a fraction 0 < *z* < 1 of the size *C* of the set of nucleotides covering the given site. If the number of bases covering the given site is denoted by *C* then *M* = *zC* is the number of observations used to compute one sample vector ***p***_*j*_ = (*p*_*j*1_,…, *p*_*jn*_) and the corresponding sample size *N* is given by (the integer part of) the logarithm of the total number of all possible sample vectors:

$$N = \left[ {\log \Upgamma \left( C \right) - \log \Upgamma \left( {zC} \right) - \log \Upgamma \left( {\left( {1 - z} \right)C} \right)} \right].$$

*Stirling’s formula* gives the following approximation in terms of *C*:

$$N \approx {{C\left( { - z\log z - \left( {1 - z} \right)\log \left( {1 - z} \right)} \right)} \mathord{\left/ {\vphantom {{C\left( { - z\log z - \left( {1 - z} \right)\log \left( {1 - z} \right)} \right)} {\log 2.}}} \right. \kern-0pt} {\log 2.}}$$

For instance, for the default value of *z*, which is 80 %, one has a sample of size *N* ≈ 0.7*C*, each sample vector computed from 0.8 *M* nucleotides. On the other hand, the value *z* = 50 % gives a sample of size *N* ≈ *C*, each sample vector computed from 0.5 *M* nucleotides.

Once the hyper-parameters of the prior distribution are estimated, they must be used together with the sequenced data of the other experimental conditions in order to compute the hyper-parameters of the posterior distributions by *Bayes’ formula*, as a result, one obtains a family of Dirichlet probability distributions indexed by the genome of the organism for every sequenced experimental condition, including the control experiment.

In order to obtain point estimates of categorical probabilities per site for each experimental condition (*p*_A_, *p*_T_, *p*_C_, *p*_G_), one may use a central tendency measure of the corresponding Dirichlet distribution (see [49]). Let ***x*** = (*x*_1_,…, *x*_*n*_) be a random vector distributed according to a Dirichlet distribution with corresponding hyper-parameters (*α*_1_,…,*α*_*n*_) then the number *s* = *α*_1_ +⋯+ *α*_*n*_ is called the *concentration parameter* of the corresponding Dirichlet distribution. It provides a measure of the “quality” of the inference: the greater the value of *s* the better is the “precision” of the inference (see [49]). The *mean value* of ***x*** is

$$\langle x_{k} \rangle = {\alpha_{k}/{s}}.$$

The *maximum* a posteriori (MAP) estimate, which is given by the *mode* of ***x***, has become a very popular method of point estimation [10]. Moreover, the coordinates ‹‹*x*_*k*_›› of the mode of ***x*** may be directly calculated in terms of the hyper-parameters *α*_*k*_ only when *α*_*k*_ > 1 (*k* = 1,…,*n*):

$$\langle\langle x_{k} \rangle\rangle \,=\, {\alpha_{k}}/(s-n).$$

This is much simpler than the contrived expectation–maximization (EM) approximate schemes usually employed to obtain the MAP estimate from a log-likelihood function, in which case approximations are unavoidable, since this function is non-convex.

Note that both the mean ‹*x*_*k*_› and the mode ‹‹*x*_*k*_›› converge to the same value when the number of observations goes to infinity. In particular, when the number of observed nucleic bases at certain site is very large then the relative frequencies of the different nucleic bases are very close to the values of the Dirichlet mean and mode.

Confidence values associated to the point estimates may be defined in terms of a dispersion measure of the corresponding Dirichlet distribution. The *variance* of ***x*** is given by

$${\mathbf{Var}}\left( {x_{k} } \right) = {\alpha_{k} {s - \alpha_{k}}}/\left({s^2(s+1)}\right) .$$

Since the marginal distribution of each *x*_*k*_ is a one-dimensional Dirichlet distribution, also known as *Beta distribution*, the *standard deviation of the mean* σ(*x*) = square-root(**Var**(*x*)) may be used to construct *Bayesian credible intervals* about the expectation value.

The the *standard deviation of the mean* σ(*x*_*k*_) may be used to define credible intervals about the mode as well. Since the Beta distribution is unimodal, when all *α*_*k*_ > 1 (*k* = 1,…, *n*), and has finite variance, a 3-sigma interval around the mean or the mode would provide about 95 % of confidence in the prediction (this is a general consequence of the Gauss-Vysochanskij-Petunin inequality, see [56]).

Finally, we should note that the inference procedure explained above is clearly not restricted to the case of four nucleotides (A, T, C, G), that is *n* = 4. It is trivial to modify it in order to account for insertions and deletions, or to work with codons and amino-acids.

### Selection criteria and error filtering

Once the inference has been completed it is desirable to filter the errors and extract some subset of the data—for instance, most conserved sites, most variable sites, etc. In order to do so we have implemented two selection criteria based on simple quantities: (1) complementary probability per site and (2) variational distance per site.

The *complementary probability per site* is defined as *p*_comp_ = 1 − max{*p*_A_, *p*_T_, *p*_C_, *p*_G_} and it depends only on the probability distribution of each site. It provides a measure of how much the distribution is concentrated in one state. For instance, if the complementary probability at a site is high it means that there was variation in the site prior to the experiment.

The *variational distance per site* is a positive number between 0 and 2 defined by $vd = \left| {p_{A} - p_{A}^{\prime } } \right| \,+\, \left| {p_{AT} - p_{T}^{\prime } } \right| \,+\, \left| {p_{C} - p_{C}^{\prime } } \right| \,+\, \left| {p_{G} - p_{G}^{\prime } } \right|$, where $\left( {p_{A} ,p_{T} ,p_{C} ,p_{G} } \right)$ is the probability distribution per site of the control experiment and $\left( {p_{A}^{\prime } ,p_{T}^{\prime } ,p_{C}^{\prime } ,p_{G}^{\prime } } \right)$ is the probability distribution at the corresponding site of the experimental condition. It is a measure of the relative variation per site from the clonal population before and after the infection. If it is very low at a site it means that the site did not undergo significant changes in relation to the sequenced data from the control experiment.

The complementary probabilities and the variational distance can work as filters and the user must specify the thresholds for them. By using these two criteria in combination one may easily obtain some qualitative information about the behavior at a site.

An example of how to combine our method with any haplotype reconstruction procedure is the following. In a haplotype reconstructed population the fraction of a nucleic base *X* at a given position could be computed by summing the proportions of all haplotypes that have the nucleic base *X* at the given position. Using these proportions one can construct a family of distributions (*f*_A_, *f*_T_, *f*_C_, *f*_G_), with *f*_A_ + *f*_T_ + *f*_C_ + *f*_G_ = 1, per position. Since in practice it is very difficult to obtain the low-frequent variants the variational distance between the distribution (*f*_A_, *f*_T_, *f*_C_, *f*_G_) and the distribution (*p*_A_, *p*_T_, *p*_C_, *p*_G_) can be used to estimate how far one is from having obtained all the variants up to the lowest-frequent ones.

Another application, is to use the distributions (*p*_A_, *p*_T_, *p*_C_, *p*_G_) to generate a population of “random haplotypes” with the correct nucleic base distribution and compare with a population of reconstructed haplotypes in order to study the correlations between the sites.

## Results and discussion

The method presented here was tested on samples obtained after the HIV-1 strain NL4-3 (group M, subtype B) cultivation on primary human cell cultures. Different viral propagation conditions were used—varying the cellular activation status, the co-receptor usage and the target cells. The pseudo-typed viruses produced in these experiments were able to perform exactly one round of the replicative cycle. As a whole, there were seven experimental conditions in addition to the *control experiment* (Table 1).

### Experimental procedure and preparation

The experimental procedure was performed in accordance with the standard procedures of the NGS platform SOLiD™ [46], up to the generation of FASTA files, which are the input data of our computational tool. Standard Life Technologies guidelines were used during sample preparation and sequencing while using the SOLiD™ platform v. 3.0. The size of the FASTA files containing the reads of each condition is around 700 Mb, consisting of about 10^7^ reads.

### Read mapping/alignment

The use of BLAST to perform the read mapping has two reasons: first the multiple reference sequences allowed by BLAST makes it advantageous for analysis of viral populations classically described as quasispecies, since we can include several variant genomes belonging to the same phylogenetic branch, and second, it is easier to control the parameters of the alignment and multi-threading inside our program.

We used a reference database composed by 1258 sequences, properly aligned, all representatives of HIV-1 epidemics, most of them belonging to major group (group M) and its subtypes A, B, C, D, F, G, H, J, K, and some of their recombinants (http://www.hiv.lanl.gov/content/sequence/HelpDocs/subtypes-more.html). Some of the sequences are of complete HIV-1 genomes while others represented virtually (more than 90 % complete) complete genomes. All sequences are available at NCBI/Genbank. The reference database also contains sequences from HIV-1 group O, SIVcpz and HIV-1 strain NL4-3 and is packaged together with the software.

Even though it is known that BLAST is not the fastest aligner when compared with next-generation aligners, we were able to achieve a reasonable speed and control by setting the parameters and implementing some optimizations. For instance, even though BLAST is capable of identifying alignments in both the forward and the reverse complementary senses, we have found that manually doing this significantly increases the retrieval of reads—we had 15 % increase in the retrieval of reads. BLAST can be sensitive if the right parameters are chosen (small word size, in particular). It can find an alignment of a 42-mer with a multiple mismatches and gaps. On the other hand, some next generation aligners may fail to find an alignment if a mismatch or gap (or more than one of these) occurs within the beginning of the read, as this portion is used as a seed. An important feature of BLAST is that all alignments are returned. If a read has 1000 alignments, 1000 alignments are reported. Another advantage is the ability to perform sub-string alignments. Next generation aligners tend to be focused on aligning the entire read length. BLAST will find an alignment and report what position within the read that the alignment start and ends. Finally, BLAST is a more sensible treatment of N’s. Some of the next-generation aligners store bases in 2-bit format. Meaning they can only internally represent A, T, C, G. The solution is to randomly assign N’s to one of the other bases, a solution that some may find imperfect.

For each experimental condition, comprising the alignment of around 10^7^ reads of 50 bases against 10^3^ HIV-reference sequences with 10^4^ sites each, we were able to map around 90 % of the reads, since the estimated fraction of reads with at least one error is around 2 %, we have achieved an almost optimal retrieval of reads.

**Fig. 2:**
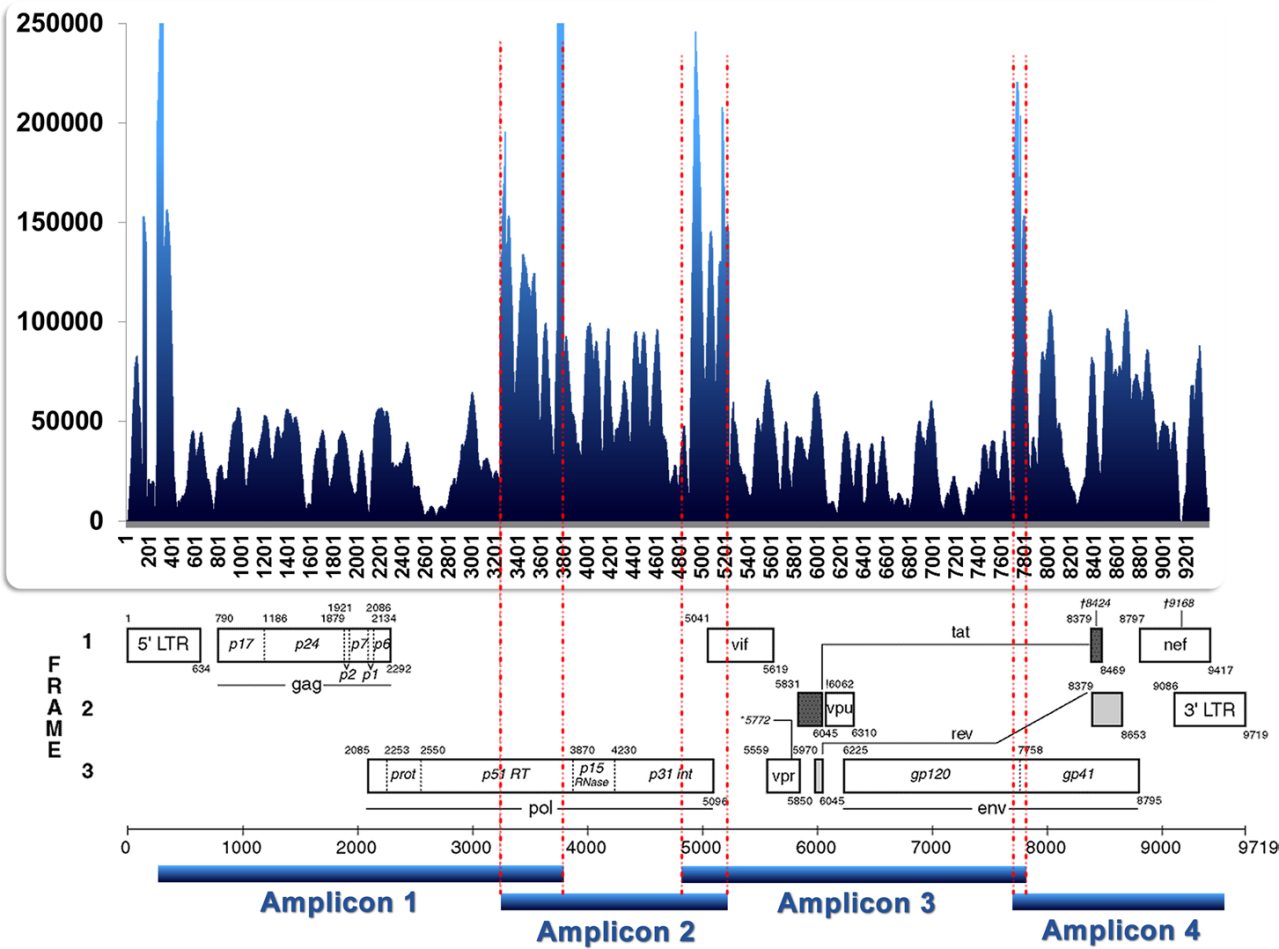
Depth and coverage of one SOLiD™ sequencing of the HIV-1 genome. The *major peaks* in the *middle* representing the most deeply covered regions coincide with the overlapping primers from the PCR step, an evidence that there is in fact some influence of pre-sequencing phases on the frequency of the short-reads retained in the alignment. The *major peak* in the *beginning* is related to the difficulties in mapping the LTR region. Other *significant peaks* maybe due to PCR artifacts, as well

For instance, Fig. 2 shows the result of the alignment of the sequenced data from the control experiment and the corresponding site coverage. The average site coverage is around 50,000 reads with some peaks going beyond 150,000 reads. The running time on each experimental condition was around 30 h on a Intel i7 (12 cores, clock of 3.30 GHz) with 32 GB of RAM memory and 2 TB of disk space. It is worthwhile mentioning that the program uses at most three cores and requires 2.8 GB of RAM memory to handle files with 700 MB, thus it is conceivable that the program could run on any computer matching this minimal configuration.

### Inference

An important difficulty that should be overcome in order to implement the inference procedure for Dirichlet hyper-parameters in the context of nucleotides is due to the *sparsity*. Even with the high mutation rate displayed by viruses, there is a fair amount of nucleotide conservation. From a populational point of view, most of individuals will present the same nucleotide at a specific genomic position, and only the less representative subgroups, if any, will present one of the three remaining possibilities.

The standard Bayesian method outlined in most textbooks, where one usually chooses an uninformative (uniform) prior distribution is appropriate for the general task of multinomial estimation [57], but generally provides poor results when used for *sparse* multinomial distributions. This is primarily a consequence of the erroneous assumption that all categories should be considered as equally possible values for each site. Indeed, sparse multinomial distributions are characterized by the fact that only a few symbols actually occur (site conservation). In such cases, applying the standard method will give too much weight to symbols that never occur and consequently give a poor estimate of the true distribution. This issue becomes critical in our case when treating data obtained from the control experiment, which, in principle, is a *clonal population*, where one expects a uniquely well-defined nucleotide at each site and thus the Dirichlet likelihood function would be identically zero.

The sparsity problem, namely, the fact that one of the categories occur with much higher frequency that the other categories, is usually solved in the literature of text modeling (see [40]) by introducing a *smoothing parameterη* and modifying the Newton–Raphson method in such a way that the sufficient statistics does not have any zero entry. In practice, this may result in an over-smoothed distribution, but one can choose a small enough value for *η* in such a way that all the rare events do not have the same probability of appearing in all states.

### Check points and validation

The method described here contains some heuristic decisions that should be supervised and properly validated. We have added a checkpoint at the read mapping stage and performed a validation of the smoothed Newton–Raphson method, attaining very good concordance with the expected results.

**Fig. 3:**
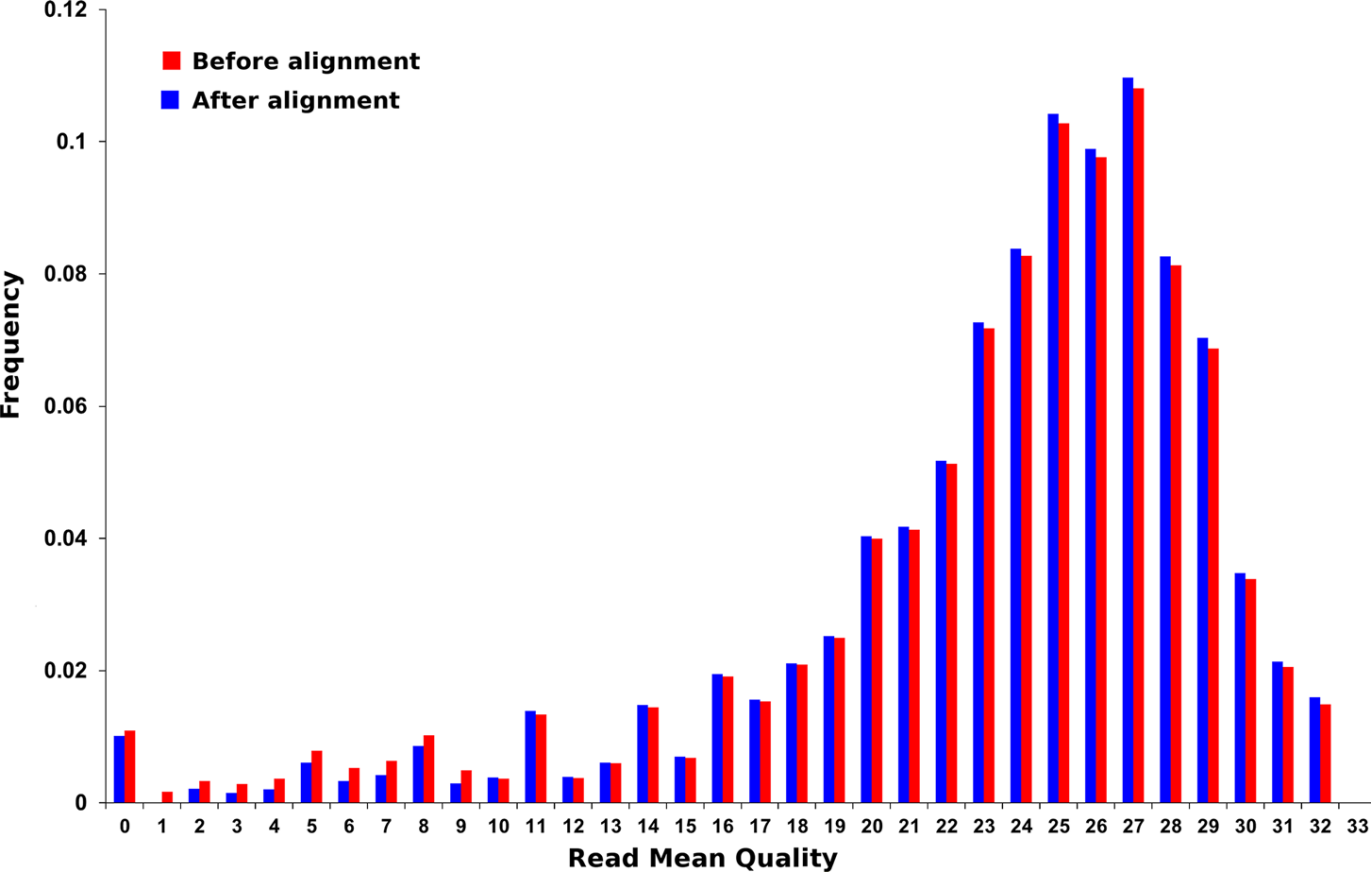
Quality values (QV) of the short-reads before (*blue*) and after (*red*) the alignment. The histogram contains the quality values on the *horizontal axis* and the proportion of short-reads in the *vertical axis*, displaying a large concentration of values around the average (approximately 23) and the majority (more than 90 %) of short-reads in the range 16–32. Short-reads with quality values in this range are considered to have excellent fidelity

In order to assess the reliability of the read mapping procedure, the *quality values* (QV) of the reads have been used as a proxy. The SOLiD™ platform outputs two files after primary analysis [46, 58]: a sequence file in color-space and a quality file containing the corresponding quality values. The QV of a read is a positive integer ranging from 0 to 50 and is given by the logarithm of the inverse probability of the color call being inaccurate, i.e. the higher the QV the higher the confidence in the color call’s accuracy. By computing the *distribution of quality values* of the reads from each experimental condition and the control experiment, prior and after the alignment, and comparing them, it is observed that they are almost identical (see Fig. 3). This shows that the alignment procedure does not introduces any bias towards higher or lower quality values. The reliability of the read mapping procedure is guaranteed by the stringency of the criteria for retaining the reads.

The checking of the read mapping procedure was done by computing the distributions of all quality values of each condition prior and after the alignment (see Fig. 3). The mean value of the QV distributions remained unchanged after alignment. Likewise, at both steps of the process more than 80 % of reads had QV comprised between 20 and 32, assuring that the quality of the retrieved sequences was preserved and no bias was introduced.

The validation of the nucleotide inference step is performed at two points. The re-sampling procedure has been validated by comparing at each site, the nucleotide frequencies obtained from all the reads that cover the site, with the nucleotide frequencies obtained from the sampled reads that cover the site. It is observed that both frequencies agree with high precision (up to order 10^−4^). This ensures that the sufficient statistics obtained with re-sampling is the correct one. The validation of the implementation of the Newton–Raphson scheme for the Dirichlet maximum likelihood is performed by computing the MLE for a standard data set that is not sparse. The data set for pollen counts analyzed in Mosiman is often used for testing Dirichlet maximum likelihood implementations (see [49]). Since our implementation has a smoothing parameter, it is expected that the obtained values converge to the known values when the parameter approaches zero. It is indeed observed that this convergence occurs, with perfect agreement occurring above the order of magnitude of the smoothing parameter.

We have included in the software the appropriate options for the user to perform validation procedures. In particular, it is possible to run the Dirichlet MLE on any data set (with 4 categories) given as a list of multinomial observations.

### Setting the smoothing parameter and separating the signal from the noise

The complementary probability of an *ideal clonal population* is expected to be identically zero. However, in the smoothed inferential scheme proposed here it is expected that *p*_comp_ ≈ *η.* The choice of smoothing parameter does not affect the running time of the Newton–Raphson method; it affects the values of the estimated hyper-parameters. The effect is of the same order of magnitude of the smoothing parameter. This suggest the following guidelines for setting the smoothing parameter: *η* must be smaller that the error rate of the sequencing. Since the expected error rate *ε* in our case is around 6 × 10^−4^ a value of *η* = 10^−5^ is a reasonable choice for the smoothing parameter.

The concentration parameter *s* of the Dirichlet distributions for the sequenced data from the control experiment is a measure of the quality of the inference: when s > 1 the inference may be considered meaningful. Sites with s ≤ 1 may be excluded from further analysis. Sites with low value of *s* may happen due poor coverage and total conservation (all reads with the same nucleotide at that position).

**Fig. 4:**
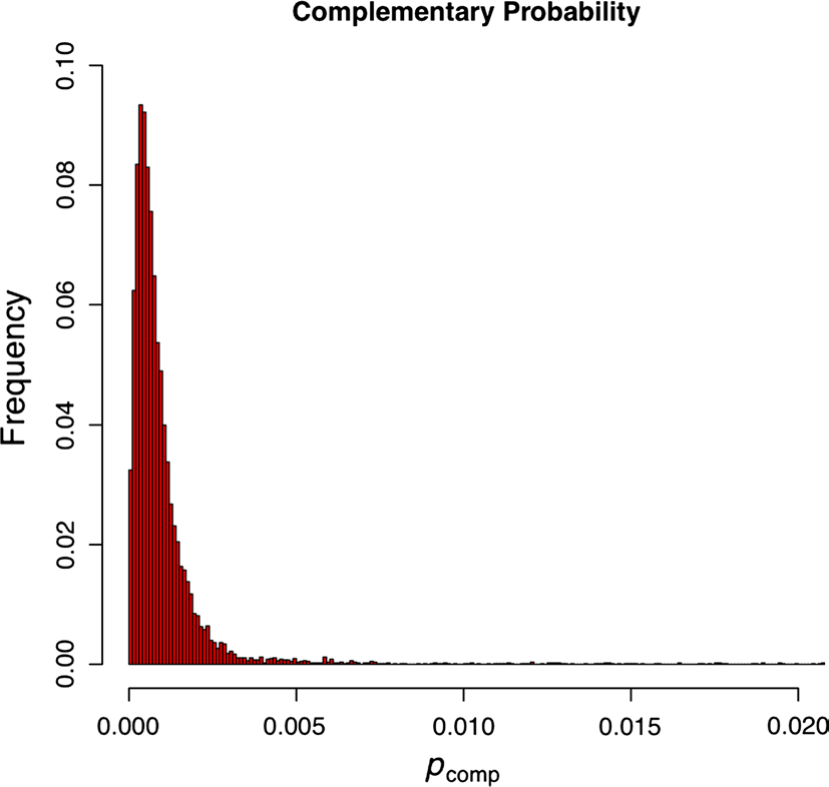
Histogram of the complementary probabilities of the control data. The complementary probability per site is defined as *p*_comp_ = 1 − max{*p*_A_, *p*_T_, *p*_C_, *p*_G_} and it depends only on the probability distribution of each site. The horizontal axis shows the values of complementary probabilities and the vertical axis the proportions of sites. The histogram contains the sites with *p*_comp_ < 0.02, which comprises 98.5 % of all genome. These are the sites that have a unique dominant nucleotide with probability greater or equal than 0.98. The remaining 1.5 % sites are the ones displaying some variability in the distribution of nucleotides

Due to sequencing and PCR errors (and other events which may have occurred prior to cloning the initial genome), *p*_comp_ may, in fact, display a broad distribution over the genome (see Fig. 4). In any case, the complementary probability of the control experiment may be considered as an average error rate per site and its distribution over the genome may be used to set a cut off value for separating the signal from the noise (everything below this value should be considered noise). It is expected that MODE(*p*_comp_) ≈ *η*, that is, the majority of sites will behave as in a clonal population and this indeed is the case (see Table 2). Furthermore, it is expected to have a concentration of the distribution of *p*_comp_ near the error rate *ε* = 6 × 10^−4^. Since the distribution of *p*_comp_ is extremely skewed with a long tail, the median is a better measure of centrality than the mean value. In fact, we have found that MEDIAN(*p*_comp_) ≈ *ε* as expected (see Table 2).

**Table 2:** Summary statistics of the complementary probability (*p*_comp_) and the concentration parameter (*s*) of the control experiment, after removal of the genomic positions with concentration parameter below 1

Statistics	*p*_comp_	*s*
Mean	0.00260	3.43
Deviation	0.01824	0.50
Median	0.00066	3.60
Mode	0.00001	3.74
Minimum	0.00001	1.01
Maximum	0.49242	6.26

The expectation MEAN(*p*_comp_), which is very sensitive to the long tail, is a reasonable conservative choice of a cut off value for noise filtering, a more conservative choice would be MEAN(*p*_comp_) + SD − MEAN(*p*_comp_). While these choices provide uniform cut off along the genome it is possible to use the individual Dirichlet distributions at each site to construct a more refined cut off function. Finally, the cut off value for *p*_comp_ can be used to obtain a cut off value for the variational distance, since the cut off value of *vd* is twice the cut off value of *p*_comp_ (*vd* is a piece-wise linear function of the probabilities).

**Fig. 5:**
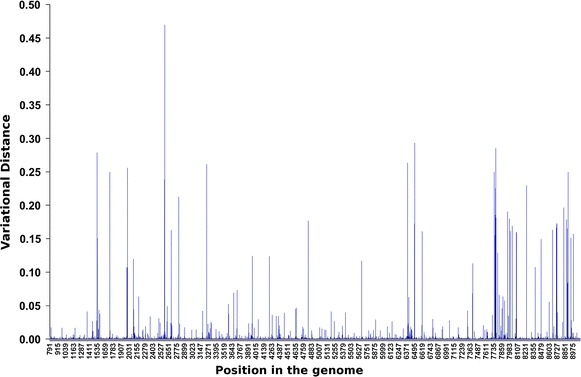
Variational distance (*vd*) between the control data and an experimental condition along the genome. The variational distance per site is defined by $vd = \left| {p_{A} - p_{A}^{\prime } } \right| + \left| {p_{T} - p_{T}^{\prime } } \right| + \left| {p_{C} - p_{C}^{\prime } } \right| + \left| {p_{G} - p_{G}^{\prime } } \right|$, where $\left( {p_{A} ,p_{T} ,p_{C} ,p_{G} } \right)$ is the probability distribution per site in the control data and $\left( {p_{A}^{\prime } ,p_{T}^{\prime } ,p_{C}^{\prime } ,p_{G}^{\prime } } \right)$ is the probability distribution of the corresponding site in the experimental condition. The *horizontal axis* shows the sites of the genome (with the LTR regions removed) and the *vertical axis* shows the corresponding variational distances. Applying the conservative cut-off value of 0.04 for *vd* one obtains the sites with significant variation

After the nucleotide probability distributions of the control experiment was computed we have found 40 genomic positions with concentration parameter *s* less or equal than one. These genomic positions correspond to portions of the genome where the coverage dropped substantially in comparison with the mean coverage (~10^2^ reads). These sites were excluded from the remaining analysis. The expectation is MEAN(*p*_comp_) = 0.002 (that is, probabilities are considered significantly distinct if they differ by more than 0.02 %). The conservative choice of cut off value is given by MEAN + SD − MEAN = 0.002 + 0.018 = 0.02 (see Table 2). The complementary probability may also be used to make sure that the variability observed in the experimental conditions is not a feature that has been transferred from the control experiment to the experimental condition. The distribution of the complementary probabilities of the control experiment shows that 98.5 % of genomic positions have *p*_comp_ < 0.02 (this means less than 2 % of nucleotide variation). The remaining 1.5 % genomic positions correspond to sites were the population acquired its variation prior to exposition to the experimental condition (see Fig. 5).

**Fig. 6:**
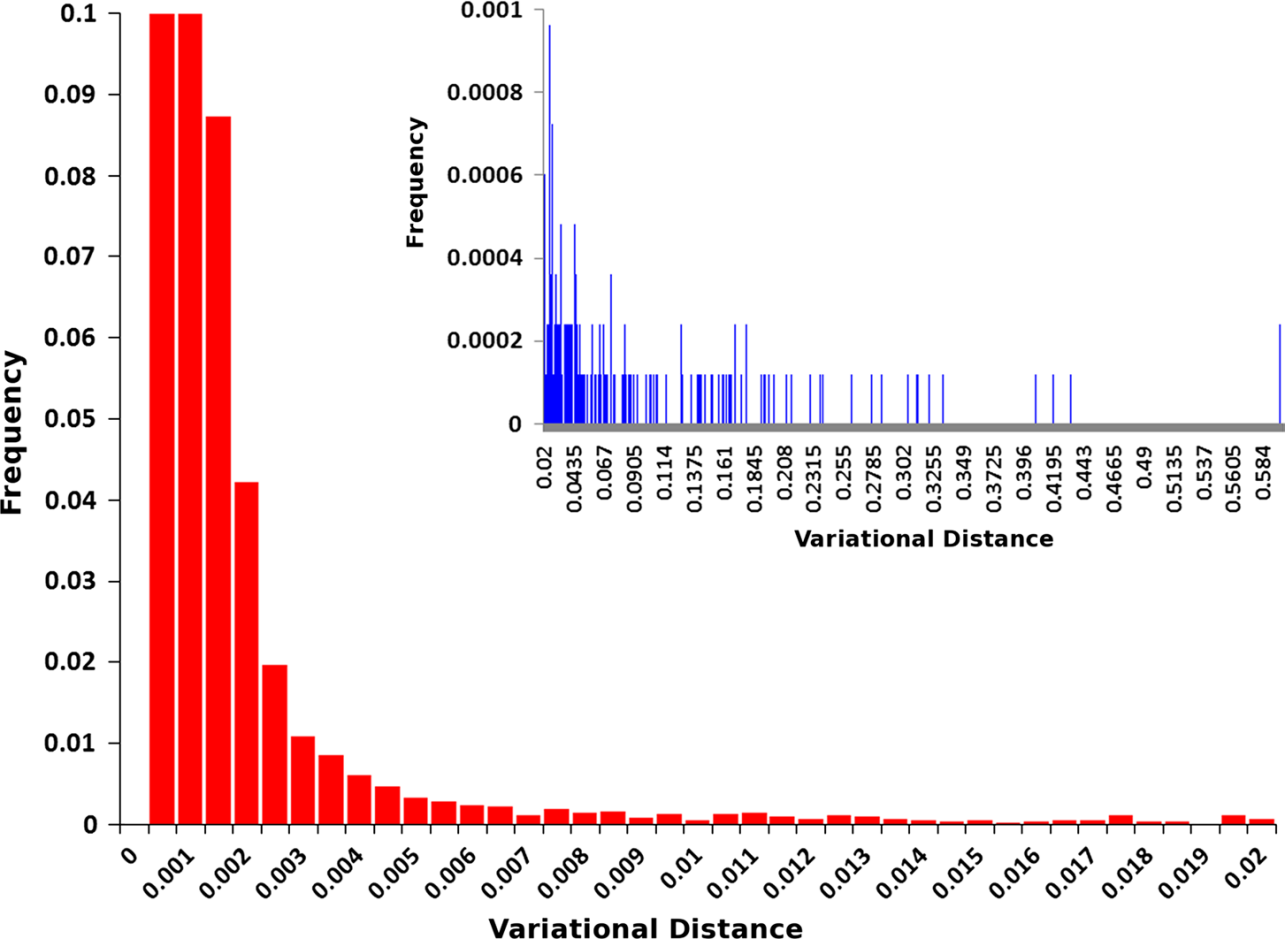
Histograms of the relative frequencies of the variational distances (*vd*). The *horizontal axis* shows the variational distances and the *vertical axis* shows the proportions of sites. The *main panel* (*red*) shows the sites with *vd* < 0.04, which comprises 98 % of all sites and the *upper panel* (*blue*) shows the sites with *vd* > 0.04, which comprises 2 % of all sites

The posterior probability distributions of all 7 experimental conditions were computed. Figure 6 presents the values of the variational distance between the control experiment and one of the experimental conditions and its distribution is shown in Fig. 6, lower panel. Considering the same conservative cut off value of 0.04 for the variational distance (Fig. 6, upper panel), one has that 98 % of the genomic positions felt under this threshold, these are sites that did not display nucleotide variation after exposition to the experimental condition. The remaining 2 % genomic positions contain all the populational variation acquired after exactly one round of the replicative cycle.

## Conclusions

High throughput sequencing technologies are constantly evolving and new platforms and refinements in the chemistry and base calling algorithms are constantly improving. Recently the PacBio™ sequencer has been gaining space as it produces long reads, but with a large number of randomly generated sequencing errors [59]. New approaches to sequencing using known technologies have been proposed, such as circle sequencing for Illumina [60]. We expect that the proposed approach, with slight modifications can be adopted for other technologies such as Ion Torrent™, Illumina™ (HiSeq, MiSeq and NextSeq) and PacBio™.

We have described a platform suitable to address the problem of estimation of populational diversity of RNA viruses. Based on the fact that the SOLiD™ sequencing platforms generate an extremely high number of reads allowing for a deep and extensive coverage of the data with very low error rate, we propose to measure the populational genetic diversity through a family of probability distributions indexed by the sites of the genome, each one representing the populational distribution of the diversity. This approach allowed us to avoid some very hard problems related to haplotype reconstruction (need of long reads, preliminary error filtering and assembly) and emphasize the main features of the sequencing technology used in this work, the SOLiD™ platform.

We have tested the method proposed here on samples obtained after the HIV-1 strain NL4-3 (group M, subtype B) cultivation on primary human cell cultures in many distinct viral propagation conditions, thus successfully demonstrating the capability of the method in handling large data-sets and delivering very clean results, suggesting that the software is a valuable tool for investigating the genetic diversity in viral populations. We have successfully demonstrated *Tanden*’s capability of handling large data-sets and delivering very clean results, suggesting that the software is a valuable tool for investigating the genetic diversity in viral populations as a complementary to some haplotype reconstruction method.

## Availability and requirements

Project name: Tanden

Project web site: http://tanden.url.ph/

Operating systems: Windows

Programming language: Microsoft-C#

License: free to all users under the LGPL license

Minimum requirements: 4 GB RAM (16 GB recommended), 500 GB disk space

Third party software used: BLAST + standalone for windows.

(http://www.ncbi.nlm.nih.gov/blast/executables/blast+/LATEST/)
